# Analysis of Physiology Theory Question Papers for Competency-Based Medical Education Implementation in Gujarat: A Pilot Study

**DOI:** 10.7759/cureus.65642

**Published:** 2024-07-29

**Authors:** Manish Ramavat, Tejas Prajapati, Pratik N Akhani, Rajesh Desai, Jitendra Patel

**Affiliations:** 1 Physiology, Gujarat Medical Education and Research Society Medical College, Vadnagar, IND; 2 Physiology, Dr. N. D. Desai Faculty of Medical Science and Research, Dharmsinh Desai University, Nadiad, IND; 3 Physiology, Gujarat Medical Education and Research Society Medical College, Patan, IND; 4 Physiology, Gujarat Medical Education and Research Society Medical College, Gandhinagar, IND

**Keywords:** faculty development, bloom's taxonomy, competency-based education, assessment, medical education

## Abstract

Introduction

Theory question papers form an important part of assessment in medical education. As per the Competency-Based Medical Education (CBME) guidelines 2019, questions should test higher levels of cognition. This pilot study analyzes 60 question papers from different universities in Gujarat for their construct and content validity. The aim was to analyze the quality of physiology question papers from various medical universities in Gujarat to gain insights into assessment quality and its alignment with the CBME guidelines. The objectives were twofold: to evaluate the "construct validity" and "content validity" of these physiology theory question papers over the past three years according to the CBME standards.

Methods

An observational study using a cross-sectional records-based approach was carried out, evaluating 60 summative exam question papers in physiology from eight different universities of Gujarat for their construct and content validity. Using Bloom’s taxonomy, the learning level of the cognitive domain for the questions asked was assessed. The findings compared and displayed a sample of papers.

Results

A total of 1842 questions were analyzed from the 60 question papers of eight different universities of the Gujarat state. The study found that the questions asked for different levels of cognition in Bloom’s taxonomy, i.e., remember, understand, apply, analyze, evaluate, and create, were 560 (30.40%), 434 (23.26%), 222 (12.05%), 118 (6.41%), 94 (5.10%), and 0.00%, respectively. A total of 414 (22.48%) questions did not have any verb, so they did not fit into any level of Bloom’s taxonomy. The majority of questions (1773, 96.25%) were asked from the core competencies, while a small percentage (69, 3.75%) of questions were asked from the non-core competencies of physiology.

Conclusion

The majority of questions in the summative question papers in physiology were of level "remember" and "understand" as per Bloom’s taxonomy. Of the questions, 26% did not have any verb. There is a need to incorporate more questions testing higher levels of cognition and to use blueprints by universities. Faculty training is also necessary to bring about course correction.

## Introduction

Assessment through theory question papers plays a very important role in any education system. When a medical university produces an Indian medical graduate (InMG), according to the National Medical Commission (NMC) guidelines [1], the graduate should be competent enough to practice medicine in society and contribute to the development of medical science. Like other sciences, an InMG needs to apply the information gained during the MBBS course in new situations and draw connections among the information when diagnosing and treating patients.

When discussing differential diagnoses with colleagues, the InMG must justify decisions based on knowledge and past experience. They must also produce new or original work based on what they have learned. As stated by the NMC - Erstwhile Medical Council of India (MCI), the assessment of an InMG should not focus on demonstrating discrete behaviors; rather, it should evaluate the application of knowledge in each patient context [1]. The United States Medical Licensing Examination (USMLE) Step 1 is a prime example of an assessment that integrates advanced cognitive abilities and case-based questions to test foundational sciences. This approach ensures that students are not only knowledgeable about basic sciences but also proficient in applying this knowledge to clinical scenarios.

Before the induction of Competency-Based Medical Education (CBME) by the NMC in 2019, many university question papers primarily contained recall-based questions or questions limited to explaining concepts, thus assessing lower cognitive levels. In 2017, Patke et al., in a retrospective study of biochemistry theory question papers, found deficiencies in the assessment of advanced cognitive abilities and in application- and case-based-type questions [2]. They also noted inconsistency and nonconformity in maintaining quality, with variations in the emphasis placed on different topics and the allocation of marks across these topics.

The term "competency-based education" refers to an outcome-based methodology that uses an organizational framework of competencies to plan, execute, assess, and evaluate medical education programs. Pass/fail choices are based on summative assessments, like university exams at the conclusion of professional courses. These tests are meant to sample learning and guarantee quality [1]. The conventional approach to expressing the utility of assessment involves representing it as a notional idea that is derived from validity, reliability, acceptability, feasibility, and educational impact [3]. The two main factors that determine the usefulness of competency-based assessment (CBA) are its impact on education and its validity. Despite subjective assessments, dependability can be raised by the use of more assessors, tasks, assessments, and departmental teachers participating during the assessment process. This straightforward strategy enhances involvement in instruction, learning, and evaluation in addition to addressing subjectivity [4]. The NMC suggests that when designing a question paper, every level of the knowledge domains, including the cognitive domain's Bloom's taxonomy, should be considered [1]. The NMC recommends that a question paper should include 20% knowledge-level questions, 20% comprehension-level questions, 20% application-level questions, 24% analysis-level questions, 8% synthesis-level questions, and 8% evaluation-level questions [1]. The person who creates the question papers needs to properly sample the information from the competencies. The NMC's guidelines emphasize the importance of assessing various levels of cognitive abilities to ensure a comprehensive evaluation of students' understanding and application of medical knowledge. Including questions across all levels of Bloom's taxonomy, ranging from simple recall to complex evaluation, question papers can better gauge the depth of students' knowledge and their ability to apply this knowledge in clinical scenarios. This approach not only aligns with modern educational theories but also prepares medical graduates to critically analyze information, solve problems, and make informed clinical decisions in their practice.

In 2022, a retrospective study by Dayanidhi et al. evaluated the content validity of exam papers that were summative in nature for the forensic medicine & toxicology subject from six medical universities in India [5]. They found the content validity unsatisfactory and emphasized the need to evaluate the effectiveness and uniformity of the university blueprints. They also highlighted the necessity of faculty training to inspire and impact a change in mindset to bring about course correction.

In the past, no similar study has been conducted for physiology question papers. Moreover, after the induction of the CBME, which clearly provides guidelines for setting up question papers, there is a need for quality assessment of theory papers to determine if universities are adhering to the CBME guidelines. In Gujarat, there is no single health university; instead, medical colleges are affiliated region-wise with respective universities. Students pursuing MBBS in Gujarat are assessed by different sets of question papers developed by the respective universities. The present study was conducted in universities of Gujarat state that have affiliated medical colleges. An analysis of final university physiology question papers from the last three academic years was performed.

The main aim of this study was to analyze the quality of physiology question papers from different medical universities in Gujarat to gain insights into the quality of assessment and its alignment with the CBME guidelines. The objectives were (a) to evaluate the "construct validity" of physiology theory question papers from the past three years, as per the CBME guidelines, from various universities in Gujarat, and (b) to evaluate the "content validity" of these question papers, also as per the CBME guidelines.

## Materials and methods

The study adopted an observational, cross-sectional, records-based approach. Ethical permission was obtained from the institute, vide letter no. GMERSMCHVAD/IEC/Research Project/approval/2023/7786 (dated: 16.10.2023). The sample comprised 60 physiology theory question papers from the years 2021 to 2023, sourced from various universities in Gujarat. Inclusion criteria encompassed physiology question papers from different Gujarat universities over the specified time frame, while physiology theory question papers from states other than Gujarat were excluded. Due to the availability of information in the public domain, explicit consent was not sought. The study was conducted as a pilot project.

Physiology papers were obtained from departmental, college libraries, or university web pages. As a pilot project, a total of eight different university question papers were collected. The number of question papers from each university varied depending on the availability. Each university's question papers showed noticeable variations. Some universities focused more on essay-type questions requiring detailed explanations, while others leaned toward multiple-choice questions for factual recall. There was variability in which topics were emphasized more prominently. For instance, one university prioritized cardiovascular physiology, while another emphasized neurophysiology. Differences were observed in how the exams were structured, such as the number and distribution of questions across different sections, the difficulty level, and the time allotted for each section.

Construct validity was assessed based on Bloom's revised taxonomy, which delineates cognitive levels corresponding to the verbs used in questions. The distribution of question levels as per Bloom's taxonomy, i.e., knowledge, comprehension, application, analysis, synthesis, and evaluation, was examined, aligning with the NMC guidelines. In India, the NMC rolled out the CBME curriculum in 2019 by replacing the traditional medical curriculum. Content validity was evaluated by mapping questions to core and non-core competencies specified in the UG-Curriculum-Vol-I by the NMC. The relevance of questions to these competencies was assessed to ensure comprehensive coverage of the construct being measured. The list of core and non-core competencies is given in UG-Curriculum Vol. I, Vol. II, and Vol. III by the NMC [1]. The percentage and frequency of questions asked at each component were calculated and evaluated.

The personnel involved in categorizing the questions according to Bloom's taxonomy were experienced medical educators and researchers. Their qualifications included advanced degrees in medical education, such as the Advance Course in Medical Education (ACME) and Foundation for Advancement of International Medical Education and Research (FAIMER), and extensive experience in curriculum development and assessment methodologies. A team of five experts was involved in the classification process. Each question was initially categorized independently by at least two experts. The initial categorizations were compared, and any discrepancies were noted. The team held meetings to discuss and resolve discrepancies. In cases where consensus was difficult to achieve, the team referred to established guidelines and examples from Bloom's taxonomy to ensure consistent and accurate classification. Only after thorough discussion and consensus was reached did the final classification of each question get documented. This rigorous process ensured that the categorization of questions was systematic, reliable, and aligned with educational standards, thus supporting the study's objective.

The frequency and percentage of questions at each cognitive level as defined by Bloom's taxonomy were calculated. Additionally, the percentages of questions pertaining to core and non-core competencies were calculated. The chi-square test was used to compare the question papers from various universities against the standard NMC norms, with a p-value of less than 0.05 considered indicative of statistical significance. The statistical software used was Microsoft Excel 2007 (Microsoft Corporation, Redmond, WA) for data entry and organization, and a trial version of IBM® SPSS® (IBM Corp., Armonk, NY) for data analysis.

## Results

A total of 60 question papers (total questions were 1842) were collected from eight universities in Gujarat state. To keep the university names anonymous, they were coded as university A to H.

We scrutinized the percentage of questions that were found at all levels of Bloom’s taxonomy of cognitive learning, which included levels such as remember, understand, apply, analyze, evaluate, and create. Data showed that the majority of questions fall under the categories of levels "Remember" and "Understand," constituting 560 (30.40%) and 434 (23.26%) of the total questions, respectively. The percentages decreased as the cognitive complexity increased, with levels "Apply," "Analyze," and "Evaluate" representing 222 (12.05%), 118 (6.41%), and 94 (5.10%) of the total questions, respectively. The question in which no verb was used comprised 414 (22.48%) of the total questions analyzed. There were no questions from the level "Create" as per Bloom’s taxonomy found in the studied questions (Table 1).

**Table 1:** University-wise distribution of questions according to Bloom’s taxonomy. A to H represents different universities. n = number of questions.

Bloom's taxonomy level	A (n = 384)	B (n = 180)	C (n = 156)	D (n = 56)	E (n = 52)	F (n = 134)	G (n = 180)	H (n = 700)	Total (A to H) (n = 1842)
No verb, n (%)	137 (35.68)	48 (26.67)	50 (32.05)	18 (32.14)	14 (26.92)	20 (14.93)	50 (27.78)	77 (11.00)	414 (22.48)
Remember, n (%)	127 (33.07)	58 (32.22)	44 (28.21)	10 (17.86)	12 (23.08)	35 (26.12)	36 (20)	238 (34.00)	560 (30.40)
Understand, n (%)	58 (15.1)	36 (20)	41 (26.28)	20 (35.71)	16 (30.77)	39 (29.1)	49 (27.22)	175 (25.00)	434 (23.26)
Apply, n (%)	23 (5.99)	18 (10)	9 (5.77)	4 (7.14)	4 (7.69)	20 (14.93)	25 (13.89)	119 (17.00)	222 (12.05)
Analyze, n (%)	27 (7.03)	11 (6.11)	6 (3.85)	2 (3.57)	3 (5.77)	11 (8.21)	9 (5)	49 (7.00)	118 (6.41)
Evaluate, n (%)	12 (3.13)	9 (5)	6 (3.85)	2 (3.57)	3 (5.77)	9 (6.72)	11 (6.11)	42 (6.00)	94 (5.10)
Total, n (%)	384 (100)	180 (100)	156 (100)	56 (100)	52 (100)	134 (100)	180 (100)	700 (100)	1842 (100)

Table 1 presents the distribution of questions according to different levels of cognition as per Bloom's taxonomy across eight universities (A to H). The highest percentage (%) of questions lacking cognitive action verbs was found in university A (36%), followed by university C (32%) and university D (32%). The lowest percentage was observed in university H (11%). University B had the highest proportion of questions at the "Remember" level (32%), closely followed by university A (33%). In contrast, university D had the lowest percentage (17%). University D featured the highest percentage of questions aimed at the "Understand" level (35%), with university E also showing a high proportion (31%). The lowest percentages were observed in university A (15%) and university B (20%). University H had the most questions at the "Apply" level (17%), while universities C, F, and G had moderate proportions ranging from 6% to 15%. The lowest percentages were in universities A (6%) and C (6%). The "Analyze" level questions were most prevalent in university F (8%) and university A (7%). Universities C, D, and G had the lowest representation at this level (3%-4%). University F had the highest percentage of questions at the "Evaluate" level (7%), followed by university G (6%) and universities B, E, and H (5%-6%). The lowest representation was seen in universities C and D (4%). This analysis demonstrates variability in the cognitive levels targeted by questions across universities, indicating different emphases on the development of cognitive skills as per Bloom's taxonomy.

Analyzing the percentage of questions sourced from core and non-core competencies, the data revealed that the vast majority of questions were derived from core competencies, accounting for 1773 (96.25%) of the total questions. Non-core competencies, on the other hand, constituted only 69 (3.75%) of the questions analyzed (Table 2).

**Table 2:** University-wise distribution of questions asked from core and non-core competencies. A to H represents different universities. n = number of questions.

University	Nature of competency	
	Core, n (%)	Non-core, n (%)
A (n = 384)	374 (98.42)	10 (2.63)
B (n = 180)	178 (98.89)	2 (1.11)
C (n = 156)	156 (100)	0 (0.00)
D (n = 56)	56 (100)	0 (0.00)
E (n = 52)	52 (100)	0 (0.00)
F (n = 134)	104 (77.61)	30 (22.39)
G (n = 180)	180 (100)	0 (0.00)
H (n = 700)	673 (96.14)	27 (3.86)
Total (n = 1842)	1773 (96.25)	69 (3.75)

Table 2 provides the distribution of questions according to core and non-core competencies across eight universities (A to H). In university A, the majority of questions (97%) focused on core competencies, with a minimal proportion of 3% addressing non-core competencies. University B had a very high concentration of questions on core competencies (99%) and a negligible focus on non-core competencies (1%). Universities C, D, E, and G exclusively concentrated on core competencies, with 100% of questions falling into this category and none addressing non-core competencies. Interestingly, university F showed a significant emphasis on non-core competencies compared to other universities, with 78% of questions on core competencies and a notable 22% on non-core competencies. University H had a high percentage of questions on core competencies (96%), with a small portion (4%) addressing non-core competencies. These data indicate that the majority of universities predominantly focus their questioning on core competencies, with university F being an outlier by incorporating a relatively higher percentage of non-core competency questions.

Table 3 compares the distribution of questions across different cognitive levels according to Bloom's taxonomy from various universities (A to H) with the norms established by the NMC. All universities exceeded the NMC norm for the "Remember" level. University H had the highest percentage (34%), followed by universities A (33%) and B (32%). The lowest was university D (17%), which was still above the norm. Most universities had a higher percentage of questions in the "Understand" category than the NMC norm. Universities D (35%) and E (31%) had notably high percentages. University A (15%) had the lowest, falling below the norm. All universities had significantly fewer questions at the "Apply" level compared to the NMC norm. University H had the highest percentage (17%), while university C (6%) had the lowest. All universities fall short of the NMC norm for the "Analyze" level. University F had the highest percentage (8%), with the lowest being university D (3%). Universities F and G approached the NMC norm with 7% and 6%, respectively, while the other universities ranged between 3% and 5%. The chi-square test results indicate that the differences between the observed distributions and the NMC norms were statistically significant, with p-values less than 0.05 for all universities. This suggests that there is a substantial deviation from the NMC norms across the universities in terms of the cognitive levels targeted by their questions.

**Table 3:** Comparison of questions from different universities against NMC norms in reference to Bloom's taxonomy level. A to H represents different universities. NMC = National Medical Commission. n = number of questions.

Blooms taxonomy level	NMC norms (%)	University							
		A (n = 384) (%)	B (n = 180) (%)	C (n = 156) (%)	D (n = 56) (%)	E (n = 52) (%)	F (n = 134) (%)	G (n = 180) (%)	H (n = 700) (%)
Remember	20	33.07	32.22	28.21	17.86	23.08	26.12	20.00	34.00
Understand	20	15.1	20.00	26.28	35.71	30.77	29.10	27.22	25.00
Apply	20	5.99	10.00	5.77	7.14	7.69	14.93	13.89	17.00
Analyze	24	7.03	6.11	3.85	3.57	5.77	8.21	5.00	7.00
Evaluate	8	3.13	5.00	3.85	3.57	5.77	6.72	6.11	6.00
P-value		0.000	0.003	0.000	0.000	0.001	0.027	0.013	0.007

## Discussion

The aim of the current study was to analyze the quality of physiology question papers of the final university examination in Gujarat state. Of the questions, 22.48% had no verb and thus did not fall into any category of Bloom’s taxonomy. Of the questions, 30.40% were of the recall level, 23.26% of the understanding level, 12.05% of the application level, 6.41% of the analysis level, 5.10% of the evaluation level, and none were of the creation level. Our results are in line with research conducted by Khuteta et al. in 2017, where they observed that the majority of the questions (92.08%) tested recollection of facts (Bloom’s taxonomy level I), with the rest (7.93%) being reasoning type questions in paper I of the pharmacology subject at a university in Gujarat [6]. Similar results were also noted by Mehta et al. in 2019 in a microbiology subject university examination, where 5% of the questions were designed to assess understanding, and the remaining 95% assessed basic knowledge in the cognitive area, with none framed to test the synthesis level of the cognitive domain [7]. Dayanidhi et al. in April 2023, while assessing the content validity of the forensic medicine and toxicology undergraduate summative examination question papers from six Indian medical universities, observed that just a single paper tested for application, and that most papers assigned 80% of the weight to recollection [5]. In 2019, Sai carried out a study on a thorough analysis of theory exam question papers of the second MBBS pharmacology subject [8]. They discovered that most of the questions (68.06%) did not meet the requirements of a standard question paper because they did not contain any verbs associated with Bloom's level of taxonomy.

After the induction of CBME in 2019, one can see that there is an improvement in construct validity in setting up the question papers. As guided by the NMC, almost all the papers had one or two case-based questions that assessed higher levels of cognition. However, a greater number of questions should be asked that evaluate higher cognitive levels to meet the standards suggested by the NMC. In addition, questions that do not have any verbs should be avoided.

The majority (96.25%) of questions were asked from core competencies only, and very few (3.75%) questions were asked from non-core competencies. Similar findings were also noted by other researchers. A study conducted by Khuteta et al. in 2017 observed that 84% of questions came from the "nice-to-know" area (8.07%), the "must-know" area (7.90%), and the "desirable-to-know" area (8.07%) in pharmacology final university question papers [6].

Mehta et al. (2019), in a microbiology subject university examination, observed that a greater proportion of questions (97%) came from the "must-know" section of the curriculum, whereas fewer questions came from the "desirable-to-know" and "nice-to-know" sections (2% and 1%, respectively) [7]. Similar findings were also noted by other researchers [9,10].

Table 3 shows that none of the universities met the standards set up by the NMC. This finding suggests that current question papers do not adequately cover the spectrum of cognitive skills necessary for a well-rounded medical education. The over-reliance on lower cognitive levels could impede the development of competencies required for effective clinical practice. The discrepancies between the observed and recommended distributions underscore the need for a systematic approach to question paper design.

The findings of this study have significant implications for medical education in Gujarat and potentially other regions in India with similar educational contexts. The predominant focus on recall-level questions suggests that students may not be adequately challenged to develop higher-order cognitive skills such as analysis, evaluation, and creation [11]. This gap underscores the need for curriculum reforms to align more closely with Bloom’s taxonomy, promoting a more comprehensive understanding and application of medical knowledge.

Implementing the NMC’s guidelines more rigorously could enhance the quality of medical education, fostering critical thinking and problem-solving abilities in future healthcare professionals. The integration of more case-based and higher-order cognitive questions can better prepare students for real-world medical practice, where such skills are crucial [12].

Furthermore, the disproportionate distribution of questions toward core competencies at the expense of non-core competencies may lead to an imbalanced education, potentially neglecting areas that are also important for well-rounded medical training. A more balanced approach to question distribution could ensure that all aspects of medical education receive appropriate attention [13].

Overall, these results emphasize the necessity of continuing assessment and reform of medical education assessments to confirm they meet contemporary educational standards and adequately prepare students for their future roles in health care. The lessons learned from Gujarat can be valuable for other regions in India and similar educational contexts globally, emphasizing the significance of continuous improvement in medical education practices.

The summative written examinations clearly placed minimal emphasis on higher cognitive domains. The content distribution was inconsistent across universities, leading to the neglect or under-representation of several topics. Validity is a fundamental aspect of effective assessment, and construct under-representation significantly threatens validity in medical education by inadequately or biasedly sampling curriculum content [9]. Blueprinting provides a strategic framework for outlining the assessment program and curriculum over a specified period. This framework helps reduce two primary threats to validity: construct under-representation (CU) and construct-irrelevant variance (CV) [14,15]. Previous studies evaluating examination papers have revealed that, although blueprinting existed, it was not consistently followed in certain specialties [9,14]. Therefore, it is recommended to establish a centralized system for the national-level moderation of examination papers to ensure consistent question distribution across different colleges [15-19]. In addition, there should be faculty training on blueprinting.

Limitations of study

The pattern of the question papers varied across different universities, with some employing short objective questions while others utilized multiple-choice questions. This variation in question formats could have influenced the results to some extent. Other significant limitations include selection bias, as the selection of universities and collection of question papers may not equally represent all institutions, potentially biasing the findings toward universities with more readily available question papers. Additionally, there is a contextual bias due to the study's exclusive focus on universities in Gujarat, which may restrict the applicability of the findings to other regions or educational systems with differing assessment methodologies. Also, our study included an analysis of all types of questions. A comparative study where question papers are separated based on their format (multiple-choice questions vs. short objective questions vs. essay-type questions) could provide deeper insights into the effectiveness of different question formats in assessing various cognitive levels and their alignment with educational objectives. To gain a more comprehensive understanding of these impacts, further research with a larger sample size is recommended. The study did not extensively explore the training and experience of faculty members who set the question papers. Differences in faculty expertise, familiarity with assessment guidelines, and pedagogical approaches could influence the quality and alignment of questions with educational objectives.

Although physiology plays a foundational role in medical education by providing essential knowledge for understanding the body's functions, its direct clinical application may be less immediate compared to subjects like medicine or surgery. Conducting a similar study on subjects such as medicine or surgery could provide insights into how these core clinical disciplines align with educational standards and cognitive domain distribution.

Recommendations for future research include expanding the study to encompass a broader and more diverse selection of universities across various states or countries. Additionally, conducting a comparative analysis of question papers utilizing different formats (such as multiple-choice questions, short objective questions, and essay-type questions) would enable an evaluation of their effectiveness in assessing diverse cognitive levels. Furthermore, exploring the influence of faculty training programs on question paper design and alignment with educational standards would provide valuable insights into improving assessment practices in higher education.

## Conclusions

This study concludes that compared to previous studies, there is an improvement in the construct validity of the summative question papers after the induction of CBME, that is after 2019. However, it does not meet the standard, expected by the NMC. There is tremendous scope of improvement in setting question paper for theory exams. None of the universities was using a blueprint for setting up the question paper for theory exams, and therefore, the use of blueprints is highly recommended. To inspire and impact a change in mindset of faculties, faculty development program should also be considered.
